# Signalment, clinical characteristics, and echocardiographic findings in dogs affected by secondary atrial fibrillation with and without concomitant frequent and complex ventricular arrhythmias

**DOI:** 10.3389/fvets.2026.1751348

**Published:** 2026-01-26

**Authors:** Giovanni Romito, Chiara Mazzoldi, Chiara Riccio, Marcello Fogli, Paola Paradies, Alessandra Recchia, Nazzareno Giuseppe Pelle, Fabio Testa, Carlotta Valente, Helen Poser, Giulia Arcuri, Barbara Contiero, Carlo Guglielmini

**Affiliations:** 1 Department of Veterinary Medical Sciences, Alma Mater Studiorum-Università di Bologna, Ozzano Emilia, Italy; 2 Department of Precision and Regenerative Medicine and Ionian Area, Section of Veterinary Clinics, University of Bari, Valenzano, Italy; 3 Independent Researcher, Bologna, Italy; 4 Clinica Veterinaria San Sebastiano, Minturno, Italy; 5 Department of Animal Medicine, Production and Health (MAPS), University of Padua, Legnaro, Italy

**Keywords:** cardiac arrhythmia, dilated cardiomyopathy, echocardiography, Holter monitoring, myxomatous mitral valve degeneration, surface electrocardiogram, ventricular premature complex

## Abstract

**Introduction:**

Assessment of dogs with atrial fibrillation (AF) and ventricular arrhythmias (VAs), two arrhythmias that can coexist, is traditionally based on 24-h Holter monitoring. However, this test may not always be feasible. The aim of this study was to determine whether alternative methods exist to distinguish dogs affected by secondary AF with frequent and complex VAs from those without.

**Methods:**

In this multicenter retrospective study, electronic databases from five centers were searched for dogs with a diagnosis of secondary AF. For inclusion, complete clinical, echocardiographic and 24-h Holter data had to be available for each dog. Signalment, clinical, and echocardiographic variables of left cardiac dimension and function were compared between dogs with secondary AF exhibiting frequent ventricular premature complexes (VPCs; ≥100/Holter) or severe VAs (Lown-Wolf grade ≥4) and those without.

**Results:**

Fifty-nine dogs, including 35/59 (59.3%) dogs with myxomatous mitral valve disease, 17/59 (28.8%) dogs with dilated cardiomyopathy, and 7/59 (11.9%) dogs with congenital heart disease, were evaluated. Compensated and decompensated cardiac disease was diagnosed in 10/59 (16.9%) and 49/59 (83.1%) dogs, respectively. Holter monitoring detected VAs associated with AF in 58/59 (98.3%) dogs. Among dogs with VPCs, the median number of VPCs was 265 (1–1,759) and 32/59 (54.2%) dogs had ≥100 VPCs/Holter. Regarding VA complexity, 34/59 (57.6%) dogs exhibited a Lown-Wolf grade ≥4. Statistical analyses revealed that none of the 17 analyzed variables reached significance in differentiating dogs with and without frequent VPCs, as well as dogs with and without complex VAs.

**Discussion:**

In dogs with secondary AF, VAs are usually present and are frequently of clinical relevance according to their Lown-Wolf grade. However, signalment, clinical presentation, and conventional echocardiographic data do not reliably distinguish dogs with frequent VPCs or complex VAs from those without, reinforcing the indispensable role of 24-h Holter monitoring for the accurate assessment of these subjects.

## 1 Introduction

In dogs, atrial fibrillation (AF) represents one of the most common cardiac rhythm disturbances and a major complication of several heart diseases, primarily myxomatous mitral valve degeneration (MMVD) and dilated cardiomyopathy (DCM) (1–5). If not promptly recognized and appropriately managed, AF can substantially increase the risk of developing congestive heart failure (CHF) and sudden death (SD) in this species (6, 7).

Accordingly, several research groups have focused on identifying factors associated with survival in dogs with AF, with the overarching aim of reducing mortality. In recent years, particular attention has been directed toward the prognostic significance of mean heart rate (mHR) in canine AF, with evidence indicating that optimized pharmacological therapy aimed at maintaining an mHR ≤ 125 beats per minute significantly prolongs survival in affected dogs (8–10). Despite appropriate rate control, however, a notable proportion of dogs with AF still experience SD (7). This observation has raised the hypothesis that additional factors may contribute to death in dogs with AF.

Recently, our research group demonstrated that dogs with secondary AF (namely AF associated with underlying structural heart disease) commonly exhibit, on 24-h Holter monitoring, a high number of ventricular premature complexes (VPCs; median: 2,606/Holter), which frequently organize into complex ventricular arrhythmias (VAs) (i.e., Lown-Wolf grade ≥4 in approximately 77% of dogs) (11). Collectively, these observations provide a rationale for hypothesizing that the relatively high prevalence of SD observed among dogs with AF and adequate mHR control may, at least in part, be attributable to under recognized, potentially fatal VAs.

Regarding VAs, accurate assessment typically depends on 24-h Holter monitoring (12–15). However, this technique is not always feasible due to its relatively high cost, the requirement for specialized equipment and technical expertise, and the level of cooperation needed from both owners and dogs. Consequently, some dogs that could benefit from Holter monitoring do not undergo the procedure for one or more of these reasons.

Because it has already been demonstrated that a short-term (≤5-min) surface electrocardiogram (ECG) is not a reliable substitute for accurately assessing VPCs count or VAs complexity in dogs with AF (11), it remains to establish whether alternative approaches can identify dogs with AF and potentially clinically significant VAs without relying on Holter monitoring.

In this context, the present study aimed to describe and systematically analyze selected variables in two populations of dogs with secondary AF—those with frequent or complex VAs and those without—to identify potential factors that may help distinguish between these groups in clinical practice, even in the absence of Holter monitoring. We hypothesized that some signalment, clinical and/or echocardiographic variables could represent reliable landmark for the presence of potentially clinically relevant VAs in dogs with secondary AF.

## 2 Materials and methods

### 2.1 Study design and animals

This retrospective observational study was conducted using clinical data retrieved from the databases of five institutions (three University Veterinary Teaching Hospitals and two private practices). For dogs included in the study, informed owner consent was obtained, which authorized the diagnostic procedures performed as well as the potential use of clinical data for research purposes.

Medical records of client-owned dogs diagnosed with secondary AF between February 2014 and February 2025 were retrospectively reviewed from the authors’ databases. To be eligible for inclusion, each dog was required to have a 24-h Holter monitoring and a complete transthoracic echocardiographic examination for diagnosis and staging of the underlying cardiac disease performed at the time of AF diagnosis. At that time, for each dog included, the following data were collected: signalment, type of structural heart disease associated with AF, disease stage according to the American College of Veterinary Internal Medicine (ACVIM) classification system (16, 17), presence and type of concurrent extra-cardiac diseases, and selected echocardiographic variables (below further details). Conversely, laboratory variables were not considered for the purposes of our analysis.

Dogs were excluded if medical records were incomplete, if 24-h Holter monitoring data were unavailable, if a diagnosis of lone AF was present, or if AF was paroxysmal.

### 2.2 Echocardiographic assessment

All echocardiographic examinations were performed by experienced operators (i.e., a board-certified cardiologist, three professors of veterinary internal medicine with more than 15 years of experience in small animal cardiology, and six veterinarians holding a PhD and/or a Master’s degree in small animal cardiology). Examinations were conducted using different ultrasound systems (iE33 and CX50, Philips Healthcare; MyLab™X7 and MyLab™X8, Esaote S.p.A.; Vivid™ iq, GE HealthCare) but followed the same standardized technique. Specifically, echocardiograms were acquired with the dogs positioned in right and left lateral recumbency in a quiet, dark room, while gently restrained and unsedated on an echocardiographic table.

Two-dimensional (2D), M-mode, and Doppler variables were measured according to established veterinary echocardiographic methods (18–20). For the purposes of this study, particular attention was paid to the following measurements: (1) left ventricular (LV) end-diastolic and end-systolic diameters, which were obtained using the 2D-guided M-mode from the right parasternal short-axis view at the level of the papillary muscles and then normalized for body weight (BW) as described by Cornell et al. (21); (2) LV end-diastolic and end-systolic volumes indexed to body surface area, which were obtained using Simpson’s method of disks from the right parasternal long-axis four-chamber view and the Teichholz method in dogs with DCM and in those with other heart diseases, respectively; (3) LV fractional shortening and LV ejection fraction, which were calculated according to standard formulas; (4) maximum anteroposterior left atrial diameter, obtained from the right parasternal long-axis four-chamber view by measuring the distance between the interatrial septum and the free wall along a line parallel to the mitral annulus at end-systole (LAD); (5) internal short-axis diameters of both the aortic diameter (Ao), which was measured along the commissure between the noncoronary and right coronary aortic valve cusps on the first frame after aortic valve closure, and the left atrial diameter (LA), which was measured in the same frame along a line extending from and parallel to the commissure between the noncoronary and left coronary aortic valve cusps to the distant left atrial margin; these measurements were then used to calculate the LA-to-aortic root ratio (LA:Ao); and (6) early transmitral peak velocity, determined by pulsed-wave Doppler from the left apical four-chamber view (19–22). All echocardiographic variables were obtained by averaging measurements from at least five consecutive beats.

### 2.3 Electrocardiographic assessment

All electrocardiographic assessments, including routine ECG and 24-h Holter recordings, were reviewed by experienced operators (G.R., C.M., P.P., C.V., H.P., C.G.). The diagnosis of AF was based on at least one of the following: (1) a 6- or 12-lead surface ECG of at least 1-min duration (Cube ECG, TouchECG, Cardioline 100 S and Cardioline Delta 1 Plus, Cardioline S.p.A.; P800, Esaote S.p.A.) or (2) a good-quality single-lead ECG obtained during echocardiographic examination (23, 24). Subsequently, 24-h Holter monitoring was performed to confirm that AF was persistent (incessant) rather than paroxysmal, identify and quantify concomitant VPCs and analyze VAs complexity. Holter recordings were acquired using different systems (Cardioline S.p.A., Cavareno; ClickHolter, Cardioline S.p.A.; Dynamic ECG Systems Digital 3-lead 24-h Analyzer Recorder System, Contec Medical Systems Co., Ltd., China), following standardized acquisition protocols (13). Specifically, recordings were obtained in the dogs’ home environment during normal daily activities, and owners were instructed to maintain a detailed activity log (13). Each recording underwent an initial manual review by the operators to assess overall quality, verify accurate software detection of complexes, and identify any unrecognized beats (13).

For the purposes of this study, manual differentiation between true VPCs and QRS complex alterations related to rate-dependent aberrancy (Ashman phenomenon) was considered essential. Differentiation was based on a detailed analysis of the following electrocardiographic criteria: (1) the coupling interval of the wide QRS complex with the previous beat (true VPCs typically have a fixed coupling interval, while the Ashman phenomenon systematically results from a long R-R/short R-R sequence); (2) the presence of a pause after the wide QRS complex (true VPCs are typically followed by a post-extrasystolic pause, which is usually absent in the Ashman phenomenon); (3) the morphology of the wide QRS complex (the configuration of true VPCs can vary within a recording due to the occurrence of VPCs arising from different ectopic foci, while QRS complex related to the Ashman phenomenon tends to show a stable morphology); and (4) the tendency of the wide QRS complex beats to form groups (VPCs may appear as couplets, triplets, or bigeminy, whereas such type of organization is atypical for the Ashman phenomenon) (13).

After VPCs were identified, their number and organization were quantified and categorized as follows: couplets (two consecutive VPCs), triplets (three consecutive VPCs), bigeminy (a VPC following every sinus beat), trigeminy (a VPC following every two sinus beats), accelerated idioventricular rhythm (≥4 VPCs at a rate of 60–180 beats/min), and ventricular tachycardia (≥4 VPCs at a rate >180 beats/min) (14, 15). Ventricular arrhythmias were further classified using a modified Lown-Wolf grading system: grade 0: no VPCs; grade 1: isolated VPCs; grade 2: ventricular bigeminy or trigeminy; grade 3: accelerated idioventricular rhythm; grade 4: ventricular couplets or triplets; and grade 5: ventricular tachycardia (14, 15). If multiple VAs types were present in the same dog, the highest degree of arrhythmic complexity was recorded for classification (e.g., if both ventricular bigeminy and tachycardia were observed, the dog was assigned grade 5) (14, 15).

### 2.4 Statistical analysis

Statistical analysis was carried out using different commercial software (MedCalc Statistical Software version 16.4.3, MedCalc Software, Ostend, Belgium; SAS 9.4, SAS Institute Inc., Cary, NC, USA; and XLSTAT version 2023.3.0, Lumivero, 2025; https://www.xlstat.com/en, accessed on 01 August 2025). Signalment and clinical characteristics included breed, sex, age, body weight, type and severity of cardiac disease, and presence of concurrent diseases. For breed and severity of cardiac disease, the following dichotomic categories were considered, respectively: purebred and crossbred; and compensated and decompensated cardiac disease (i.e., B1 + B2 and C + D stage of the ACVIM classification system, respectively). The following continuous echocardiographic variables were considered: LV end-diastolic and end-systolic diameters; LV end-diastolic and end-systolic diameters normalized for BW; LV end-diastolic and end-systolic volumes indexed to body surface area; LV fractional shortening and ejection fraction; LAD; LA; LA:Ao; and early transmitral peak velocity.

Data distribution was assessed using the Shapiro–Wilk test. Continuous clinical and echocardiographic variables showed a normal distribution and were therefore reported as mean ± standard deviation. In contrast, the number of VPCs recorded on Holter monitoring did not follow normal distribution and was consequently reported as median (minimum–maximum range). Categorical variables were presented as number and percentage within each category. The comparison of signalment, clinical and echocardiographic variables between dogs with and without ≥100 VPCs/Holter or Lown-Wolf grade ≥4 was carried out using the Student’s *t*-test and the two-proportion *z*-test for continuous and categorical variables, respectively. The aforesaid frequency threshold was selected because occasional VPCs may occur in healthy adult dogs, whereas exceeding 100 VPCs within 24 h is uncommon and likely reflects a non-random, pathological phenomenon (25–29). The second criterion was based on the Lown-Wolf classification, as higher grades are associated with increased cardiac mortality both in human patients (30) and in dogs (31, 32).

For all analyses, the significance was set to *p* < 0.05. Since no variables showed statistically significant differences between the two groups of dogs in the univariate analyses, and no clinically meaningful trends or relevant effect sizes were identified, no further analyses were performed (e.g., univariate screening followed by stepwise multivariable logistic regression to estimate the odds ratio for the presence of numerous VPCs or complex VAs). Given these findings, multivariate modeling was considered unlikely to provide additional interpretative value and could have increased the risk of overfitting.

## 3 Results

A total of 59 dogs with secondary AF met the inclusion criteria, including 43/59 (72.9%) males and 16/59 (27.1%) females. Most dogs were crossbred (15/59 [25.4%]). Purebred dogs included German Shepherd (7/59 [11.9%]), Dogue de Bordeaux (6/59 [10.2%]), Weimaraner (4/59 [6.8%]), Dachshund and Golden Retriever (3/59 [5.1%] each), Corso, Doberman Pinscher, Maremmano-Abruzzese Sheepdog and Miniature Pinscher (2/59 [3.4%] each), as well as American Bulldog, American Staffordshire Terrier, Appenzeller Sennenhund, Beagle, English Springer Spaniel, Giant Schnauzer, Jack Russell Terrier, Leonberger, Newfoundland, Pointer, Rottweiler, Spinone Italiano and Vizsla (1/59 [1.7%] each). The mean age was 10 ± 4 years and the mean BW was 32 ± 17 kg.

Regarding underlying heart disease, 35/59 (59.3%) dogs had MMVD, 17/59 (28.8%) had DCM and 7/59 (11.9%) had congenital heart disease, including three cases of isolated tricuspid dysplasia, one case of isolated mitral dysplasia, one case of combined mitral and tricuspid dysplasia, one case of patent ductus arteriosus and one case of ventricular septal defect. At the time of inclusion, 10/59 (16.9%) dogs were classified as having compensated heart disease (ACVIM stage B2), whereas 49/59 (83.1%) had decompensated disease, including 45 cases at ACVIM stage C and 4 cases at ACVIM stage D.

Extra-cardiac diseases were documented in 19/59 (32.2%) dogs. These conditions included oncologic (5/19 [26.3%]; chemodectoma, cutaneous hemangiosarcoma, cutaneous mast cell tumor, glioma and osteosarcoma [1 each]), endocrine (4/19 [21.1%]; hypothyroidism [2], hyperadrenocorticism and hypoadrenocorticism [1 each]), gastrointestinal (3/19 [15.8%]; food-responsive enteropathy [3]), nephrological (2/19 [10.5%]; stage II chronic kidney disease and urolithiasis [1 each]), respiratory (2/19 [10.5%]; tracheobronchial collapse in both cases), orthopedic (2/19 [10.5%]; hip dysplasia, chronic osteoarthritis [1 each]) and dermatologic (1/19 [5.3%]; chronic allergic dermatitis) diseases.

Holter monitoring detected VAs associated with AF in 58/59 (98.3%) dogs. Among dogs with VPCs, the median number of VPCs was 265 (minimum–maximum range 1–1,759). Of these, 32/58 (55.2%) dogs had ≥100 VPCs on Holter recording, with a median of 1,417 (585–3,436). Regarding VAs complexity, 34/59 (57.6%) dogs showed a Lown-Wolf grade ≥4. Specifically, 1/59 (1.7%) dog had grade 0, 16/59 (27.1%) had grade 1, 7/59 (11.9%) had grade 2, 1/59 (1.7%) had grade 3, 23/59 (39.0%) had grade 4, and 11/59 (18.6%) had grade 5. Antiarrhythmic treatment protocols included: dual therapy with diltiazem and digoxin (27/59 [45.8%] dogs); digoxin monotherapy (13/59 [22.0%] dogs); diltiazem monotherapy (6/59 [10.2%] dogs); amiodarone monotherapy (6/59 [10.2%] dogs); triple therapy with amiodarone, digoxin, and diltiazem (3/59 [5.1%] dogs); dual therapy with amiodarone and digoxin (1/59 [1.7%] dog); and dual therapy with amiodarone and diltiazem (1/59 [1.7%] dog). In the remaining 2/59 (3.4%) dogs, no antiarrhythmic drugs were initially prescribed given the relatively low mHR, number of VPCs, and complexity of VAs.

According to statistical analyses, no significant differences were found when comparing selected signalment, clinical and echocardiographic variables between dogs with and without frequent VPCs on Holter monitoring (Table 1). Similarly, no significant differences were observed between dogs with and without complex VAs (Table 2).

**Table 1:** Descriptive data obtained from 59 dogs with atrial fibrillation classified in two groups based on the number of ventricular premature complexes detected on 24-h Holter monitoring.

Variable	Category	Total (*N* = 59)	≥100 VPCs/Holter (*N* = 32)	<100 VPCs/Holter (*N* = 27)	*p* value
		Number (%), mean ± SD	Number (%), mean ± SD	Number (%), mean ± SD	
Breed	Crossbreed	15 (25%)	11 (34%)	4 (15%)	0.136
	Purebred	44 (75%)	21 (66%)	23 (85%)	
Sex	F	16 (27%)	10 (31%)	6 (22%)	0.624
	M	43 (73%)	22 (69%)	21 (78%)	
Age (years)		10 ± 4	10 ± 3	9 ± 4	0.179
BW (kg)		32 ± 17	32 ± 16	31 ± 22	0.873
Heart disease	CHD	7 (12%)	2 (6%)	5 (19%)	0.304
	DCM	17 (29%)	9 (28%)	8 (30%)	0.999
	MMVD	35 (59%)	21 (66%)	14 (51%)	0.417
ACVIM	B1 + B2	10 (17%)	4 (13%)	6 (22%)	0.524
	C + D	49 (83%)	28 (87%)	21 (78%)	
Concurrent diseases	Yes	19 (32%)	9 (28%)	10 (37%)	0.653
	No	40 (68%)	23 (72%)	17 (63%)	
LVIDd (mm)		50.71 ± 12.33	51.51 ± 9.05	49.8 ± 15.42	0.610
LVIDdN		1.94 ± 0.37	1.95 ± 0.31	1.94 ± 0.44	0.943
LVIDs (mm)		35.74 ± 12.54	36.54 ± 10.76	34.82 ± 14.49	0.613
LVIDsN		1.34 ± 0.34	1.36 ± 0.33	1.32 ± 0.37	0.612
EDVi (mL/m^2^)		137.43 ± 60.91	135.79 ± 49.25	139.33 ± 73.09	0.831
ESVi (mL/m^2^)		62.62 ± 36.22	62.26 ± 32.78	63.03 ± 40.49	0.938
FS%		29.8 ± 12.37	30 ± 12.29	29.58 ± 12.71	0.900
EF%		54.04 ± 17.34	54.7 ± 17.19	53.28 ± 17.83	0.763
LAD (mm)		56.96 ± 16.15	61.13 ± 12.83	52.29 ± 18.48	0.102
LA (mm)		50.41 ± 12.67	51.83 ± 9.35	48.77 ± 15.70	0.372
LA:Ao		2.22 ± 0.52	2.22 ± 0.45	2.22 ± 0.59	0.974
E (cm/s)		129.9 ± 37.43	136.04 ± 38.39	121.71 ± 35.34	0.188

Data are expressed as mean ± standard deviation (SD) and number (*N*) and (percentage). ACVIM, American College of Veterinary Internal Medicine; BW, body weight; CHD, congenital heart disease; DCM, dilated cardiomyopathy; E, early transmitral peak velocity; EDVi, left ventricular end-diastolic volume indexed; ESVi, left ventricular end-systolic volume indexed; FS, fractional shortening; LA, left atrial diameter measured in short axis view; LA:Ao, left atrial-to-aortic root ratio; LAD, maximum anteroposterior left atrial diameter measured in long axis view; LVIDd, left ventricular end-diastolic diameter; LVIDdN, left ventricular end-diastolic diameter normalized to body weight; LVIDs, left ventricular end-systolic diameter; LVIDsN, left ventricular end-systolic diameter normalized to body weight; MMVD, myxomatous mitral valve degeneration; VPCs, ventricular premature complexes. EF, ejection fraction; F, female; M, male.

**Table 2:** Descriptive data obtained from 59 dogs with atrial fibrillation classified in two groups based on the severity of ventricular arrhythmias detected on 24-h Holter monitoring according to the Lown-Wolf grading system.

Variable	Category	Total (*N* = 59)	≥4 Lown-Wolf grade (*N* = 34)	<4 Lown-Wolf grade (*N* = 25)	*p* value
		Number (%), mean ± SD	Number (%), mean ± SD	Number (%), mean ± SD	
Breed	Crossbreed	15 (25%)	11 (32%)	4 (16%)	0.236
	Purebred	44 (75%)	23 (68%)	21 (84%)	
Sex	F	16 (27%)	12 (35%)	4 (16%)	0.150
	M	43 (73%)	22 (65%)	21 (84%)	
Age (years)		10 ± 4	10 ± 3	10 ± 4	0.890
BW (kg)		32 ± 17	32 ± 15	31 ± 23	0.766
Heart disease	CHD	7 (12%)	4 (12%)	3 (12%)	0.999
	DCM	17 (29%)	9 (26%)	8 (32%)	0.864
	MMVD	35 (59%)	21 (62%)	14 (56%)	0.860
ACVIM	B1 + B2	10 (17%)	5 (15%)	5 (20%)	0.856
	C + D	49 (83%)	29 (85%)	20 (80%)	
Concurrent diseases	Yes	19 (32%)	10 (29%)	9 (36%)	0.801
	No	40 (68%)	24 (71%)	16 (64%)	
LVIDd (mm)		50.71 ± 12.33	50.48 ± 11.33	51.04 ± 13.9	0.869
LVIDdN		1.94 ± 0.37	1.87 ± 0.33	2.05 ± 0.41	0.068
LVIDs (mm)		35.74 ± 12.54	36.67 ± 11.54	34.4 ± 14.01	0.512
LVIDsN		1.34 ± 0.34	1.34 ± 0.34	1.34 ± 0.36	0.953
EDVi (mL/m^2^)		137.43 ± 60.91	125.47 ± 49.8	154.59 ± 71.74	0.078
ESVi (mL/m^2^)		62.62 ± 36.22	61.72 ± 32.03	63.9 ± 42.24	0.827
FS%		29.8 ± 12.37	28.45 ± 11.72	31.74 ± 13.28	0.333
EF%		54.04 ± 17.34	52.33 ± 16.03	56.49 ± 19.17	0.382
LAD (mm)		56.96 ± 16.15	58.43 ± 14.44	54.64 ± 18.86	0.500
LA (mm)		50.41 ± 12.67	51.33 ± 10.47	49.09 ± 15.46	0.519
LA:Ao		2.22 ± 0.52	2.22 ± 0.44	2.22 ± 0.62	0.958
E (cm/s)		129.9 ± 37.43	130.1 ± 39.09	129.56 ± 35.48	0.961

Data are expressed as mean ± standard deviation (SD) and number (*N*) and (percentage). ACVIM, American College of Veterinary Internal Medicine; BW, body weight; CHD, congenital heart disease; DCM, dilated cardiomyopathy; E, early transmitral peak velocity; EDVi, left ventricular end-diastolic volume indexed; ESVi, left ventricular end-systolic volume indexed; FS, fractional shortening; LA, left atrial diameter measured in short axis view; LA:Ao, left atrial-to-aortic root ratio; LAD, maximum anteroposterior left atrial diameter measured in long axis view; LVIDd, left ventricular end-diastolic diameter; LVIDdN, left ventricular end-diastolic diameter normalized to body weight; LVIDs, left ventricular end-systolic diameter; LVIDsN, left ventricular end-systolic diameter normalized to body weight; MMVD, myxomatous mitral valve degeneration; EF, ejection fraction; F, female; M, male.

## 4 Discussion

This study aimed to compare two distinct populations of dogs affected by secondary AF—namely, those with frequent VPCs or complex VAs, and those without—in order to determine whether it is possible, in clinical practice, to distinguish between them even in the absence of 24-h Holter monitoring.

The results presented herein suggest that, based on signalment, clinical presentation, or standard echocardiographic evaluation, it is not possible to accurately differentiate between the two aforementioned populations. Consistent with these findings, it has been recently demonstrated that even a short (≤5-min) ECG is not a reliable alternative to 24-h Holter monitoring for identifying the presence of frequent VPCs or severe VAs in dogs with secondary AF (11). Overall, these data confirm the indispensability of 24-h Holter monitoring for an accurate and comprehensive assessment of dogs affected by secondary AF. Indeed, this diagnostic tool is essential not only for analyzing the mHR before and after the administration of drugs aimed at rate control (e.g., diltiazem, digoxin) (8–10), but also for detecting the presence of VAs that could benefit from the implementation of antiarrhythmic therapy (e.g., amiodarone, mexiletine) (14, 15).

Another noteworthy finding of the present study concerns the prevalence and complexity of VAs. It is particularly interesting to observe that almost all dogs with secondary AF included in this study (approximately 98%) exhibited VPCs on 24-h Holter monitoring. Additionally, more than half of the population had ≥100 VPCs/Holter (approximately 55%) as well as presented with a Lown-Wolf grade ≥4 (approximately 58%). These data are overall consistent with those reported in a previous study on 142 dogs with AF by Borgeat et al. (7). Indeed, in that study sample, a high median number of VPCs (i.e., 1,591/Holter) was reported, with approximately 70% of subjects experiencing VAs of Lown-Wolf grade ≥4 (7). Moreover, in a Holter analysis on 53 dogs with different tachyarrhythmias, the coexistence of secondary AF and VAs was identified in approximately one-third of subjects (18/53 [34%]), with a median VPC count of 982/Holter (13).

Several anatomical and functional substrates may help explain why AF and VAs can coexist, particularly in dogs with MMVD and DCM. In MMVD, progressive mitral regurgitation over time leads to LA remodeling characterized by dilation and fibrosis, ultimately predisposing to the development of AF (4, 33–36). Concurrently, structural remodeling also affects the left ventricle, occurring both macroscopically and microscopically. Specifically, VAs in dogs with advanced MMVD have been associated with intramyocardial arterial narrowing and myocardial interstitial fibrosis (37). In DCM, LA dilation—which subsequently promotes AF development—is not primarily the result of mitral regurgitation but rather of impaired LV contractility. Indeed, this dysfunction increases LV end-diastolic and LA pressures, leading to progressive dilation of the left-sided cardiac chambers (5, 38). Interestingly, in this cardiomyopathy, VPCs may occur even during the occult phase of the disease and are attributed to myocardial fibrosis as well as to abnormalities in gap junctions and ion channels, which are intrinsically favored by specific genetic mutations (25–27). It should also be noted that in both MMVD and DCM, the risk of developing AF, as well as the frequency and/or severity of VAs, may further increase when the disease progresses to CHF (4, 5, 17, 34, 38). Consistent with this, the vast majority of dogs in the present study had already developed CHF prior to the diagnosis of AF, or the first episode of CHF was diagnosed on the same day as AF onset (i.e., approximately 83% of dogs were classified as ACVIM stage C or D).

Regardless of the underlying arrhythmic mechanisms, the above-mentioned findings reinforce the concept that dogs affected by secondary AF are usually also affected by concomitant VAs, which may often be of considerable clinical relevance. Accordingly, we strongly believe that, contrary to what has traditionally been assumed until recently, an updated and evidence-based approach to the management of dogs with secondary AF should no longer be limited to the control of mean mHR. Instead, it should also encompass the identification, classification, and, when indicated, treatment of concurrent VAs, with the ultimate goal of reducing the incidence of SD.

The results of this study should be interpreted in light of several limitations. First, its retrospective design precluded the standardization of diagnostic procedures, as different machines and operators were involved. However, given the standardized protocols used for cardiac assessments and the expertise of the operators performing them, it is unlikely that these factors introduced significant bias into our findings. Second, the criteria used to analyze VAs warrant careful consideration when interpreting our findings. Of note, two parameters were purposefully applied, namely VPC frequency (i.e., ≥100 VPCs/Holter) and arrhythmic organization (i.e., Lown-Wolf grade ≥4). In our opinion, using both parameters provided a more comprehensive assessment of arrhythmic picture than either alone. However, while the selected cut-offs are evidence-based and likely clinically relevant, applying different Holter thresholds could yield alternative results. Regarding the Lown-Wolf grading system, it was originally developed to evaluate the risk of death from ischemic heart disease in humans, rather than in dogs with arrhythmias secondary to conditions such as MMVD and DCM. However, it should be acknowledged that this system has been widely applied in veterinary cardiology for more than two decades (10–12, 14, 15), as the pathophysiological sequelae associated with VAs are believed to be similar in humans and dogs. Third, the study population was heterogeneous in terms of both breed and underlying cardiac disease. However, this diversity strengthens the external validity of the findings, reflecting real-world clinical scenarios in which dogs of various breeds and cardiac profiles are routinely evaluated. Conversely, restricting the analysis to a single breed or disease (e.g., only Cavalier King Charles Spaniels with MMVD) would have limited the broader clinical applicability of the results. Fourth, the lack of statistically significant difference between groups should be interpreted with caution. Although the observed results may suggest an absence of clear group-related effects, the relatively small sample size limited the statistical power of the study and precluded the use of multivariate analyses. Therefore, it cannot be excluded that subtle or combined effects of multiple variables may have gone undetected. Negative findings should thus be considered in the context of these methodological constraints rather than interpreted as definitive evidence of true equivalence between groups. Fifth, although some laboratory changes may affect electrocardiographic findings in dogs (e.g., severe electrolyte imbalances), these were not considered in the statistical analysis, primarily because blood work performed on the day of the cardiologic examination was not available for all dogs. Sixth, the effect of antiarrhythmic drugs on the number of VPCs and complexity of VAs was not assessed. This should be considered as some of the drugs employed in the study populations could have reduced the VPCs number and VAs complexity [e.g., amiodarone (14)], while others could have enhanced them [e.g., digoxin (10)]. Lastly, in this study, echocardiographic assessment primarily focused on the morphology and function of the left cardiac chambers, whereas parameters related to right heart were not included in the statistical analysis. This decision was based not only on the fact that complete right-sided echocardiographic data were unavailable for all dogs, but also because the parameters selected for our analysis are those with well-established diagnostic and prognostic significance [e.g., increased LA dimensions in MMVD (16, 39) and reduced LV systolic function in DCM (17)]. Nevertheless, we cannot entirely exclude the possibility that the relatively limited number of echocardiographic variables considered may have hindered the identification of factors capable of distinguishing between the two study populations. This is particularly relevant considering that, for example, VAs have been shown to influence right heart echocardiographic parameters in humans (40, 41). Moreover, it should be noted that only conventional echocardiographic techniques (i.e., M-Mode, two-dimensional, and Doppler evaluation) were used in our analysis. Therefore, it remains to be determined whether the application of advanced modalities previously employed in studies of canine supraventricular tachyarrhythmias, including AF [e.g., Tissue Doppler Imaging (42, 43), Speckle Tracking Echocardiography (3)], could aid in differentiating the two populations compared in this study.

## 5 Conclusion

In conclusion, this study demonstrates that VAs are extremely common in dogs with secondary AF, are frequently of considerable clinical relevance according to their Lown-Wolf grade, and that neither signalment, clinical presentation, nor conventional echocardiographic parameters can reliably distinguish dogs with secondary AF that exhibit frequent VPCs or severe VAs from those without. Taken together, these findings underscore the essential role of 24-h Holter monitoring in the comprehensive evaluation of dogs with secondary AF—an approach that should go beyond mHR assessment to include the identification, staging, and, when appropriate, management of concurrent VAs. Further studies are warranted to determine whether the presence and frequency of VPCs, as well as the complexity of VAs, influence survival in dogs with secondary AF.

## Data Availability

The raw data supporting the conclusions of this article will be made available by the authors without undue reservation.
